# A pilot study on using rapamycin-carrying synthetic vaccine particles (SVP) in conjunction with enzyme replacement therapy to induce immune tolerance in Pompe disease

**DOI:** 10.1016/j.ymgmr.2017.03.005

**Published:** 2017-07-23

**Authors:** Han-Hyuk Lim, Haiqing Yi, Takashi K. Kishimoto, Fengqin Gao, Baodong Sun, Priya S. Kishnani

**Affiliations:** a Division of Medical Genetics, Department of Pediatrics, Duke University Medical Center, Durham, NC, United States; b Selecta Biosciences, Inc., Watertown, MA, United States

**Keywords:** ERT, enzyme replacement therapy, rhGAA, recombinant human acid-α-glucosidase, CRIM, cross-reactive immunologic material, HSAT, high and sustained antibody titer, MTX, methotrexate, SVP-Rapa, synthetic vaccine particles carrying rapamycin, NP, empty nanoparticles, Pompe disease, Acid alpha-glucosidase, Enzyme replacement therapy, Tolerogenic nanoparticles, Rapamycin

## Abstract

A major obstacle to enzyme replacement therapy (ERT) with recombinant human acid-α-glucosidase (rhGAA) for Pompe disease is the development of high titers of anti-rhGAA antibodies in a subset of patients, which often leads to a loss of treatment efficacy. In an effort to induce sustained immune tolerance to rhGAA, we supplemented the rhGAA therapy with a weekly intravenous injection of synthetic vaccine particles carrying rapamycin (SVP-Rapa) during the first 3 weeks of a 12-week course of ERT in GAA-KO mice, and compared this with three intraperitoneal injections of methotrexate (MTX) per week for the first 3 weeks. Empty nanoparticles (NP) were used as negative control for SVP-Rapa. Co-administration of SVP-Rapa with rhGAA resulted in more durable inhibition of anti-rhGAA antibody responses, higher efficacy in glycogen clearance in skeletal muscles, and greater improvement of motor function than mice treated with empty NP or MTX. Body weight loss was observed during the MTX-treatment but not SVP-Rapa-treatment. Our data suggest that co-administration of SVP-Rapa may be an innovative and safe strategy to induce durable immune tolerance to rhGAA during the ERT in patients with Pompe disease, leading to improved clinical outcomes.

## Highlights

• SVP-Rapa induced durable rhGAA-specific immune tolerance to ERT in GAA-KO mice.
• SVP-Rapa co-treatment reduced muscle glycogen more efficiently than methotrexate.
• SVP-Rapa significantly improved muscle strength and motor function by ERT.
• This study suggests an innovative and safe immunomodulatory ERT for Pompe disease.

## 1 Introduction

Pompe disease (glycogen storage disease type II, OMIM 232300) is a lysosomal storage disorder caused by a deficiency of lysosomal enzyme acid-α-glucosidase (GAA; acid maltase; EC 3.2.1.20), and characterized by progressive structural disruption and cell dysfunction of muscle tissues due to lysosomal accumulation of glycogen [1]. Without treatment in classic infantile Pompe disease, which represents the most severe end of the disease spectrum, death secondary to cardiorespiratory failure typically occurs within the first 1–2 years of life [2], [3]. The availability of intravenous enzyme replacement therapy (ERT) with recombinant human acid-α-glucosidase (rhGAA, alglucosidase alfa, Myozyme®) has dramatically improved overall survival and daily activities for patients with Pompe disease [4], [5]. However, the development of high and sustained antibody titer (HSAT) against the therapeutic rhGAA occurs in cross-reactive immunologic material negative (CRIM-) patients and a subset of CRIM + patients, which severely compromises the safety and efficacy of the ERT [6], [7]. Patients with HSAT respond poorly to ERT and need an additional immunomodulation therapy to prevent ongoing disease progression [6], [8]. A broad range of agents have been evaluated for immune tolerance induction, among which rituximab (monoclonal anti-CD 20), rapamycin, mycophenolate mofetil, cyclophosphamide, belimumab (anti-B-cell activating factor; anti-BAFF), Methotrexate (MTX), intravenous immunoglobulin (IVIG), and bortezomib have been shown to be capable of modulating the anti-rhGAA antibody response [9], [10], [11], [12], [13]. However, these universal immunosuppressant agents induce systemic immune suppression and may cause side effects such as bone marrow and gastrointestinal toxicities with the possibility of opportunistic infections and tumorigenesis, and chronic administration is often needed in those with an established immune response [10], [11], [14].

For immune tolerance induction in diseases treated with immunogenic drugs, it would be desirable to transiently target the immunosuppressant's effects to dendritic cells and other antigen-presenting cells at the time of antigen encounter. Dendritic cells play a key role in antigen presentation to helper T-cells and control of the immune response [15]. Synthetic vaccine particles (SVP™), also called nanoparticles (NP), effectively deliver antigen and drug to antigen-presenting cells in a similar way as a virus [16]. Recently, Maldonado et al. used nanoparticle-encapsulated antigen together with rapamycin, a tolerogenic immunomodulator, to induce immunological tolerance in hemophilia A mice [17]. They demonstrated that NP containing both the immunosuppressant rapamycin and an antigen (coagulation factor VIII) inhibited antigen-specific CD4 + and CD8 + T-cell activation, increased regulatory cells, induced durable B-cell tolerance, and inhibited antibody responses against coagulation factor VIII. Subsequently, two studies reported that co-administration of free antigen and SVP containing rapamycin (SVP-Rapa) induced antigen-specific and SVP-Rapa-dependent immune tolerance in mice and non-human primates [18], [19]. In this study, we demonstrate that SVP-Rapa can induce immune tolerance to rhGAA and improve efficacy of ERT in GAA-knockout (KO) mice that is superior to immunosuppression with MTX.

## 2 Material and methods

### 2.1 Drugs

The rhGAA (Myozyme®, alglucosidase alfa; manufactured by Sanofi Genzyme) was purchased from Pharmaceutical Buyers, Inc. (New Hyde Park, NY). Empty NP and SVP-Rapa were prepared and provided by Selecta Biosciences, Inc. (Watertown, MA, USA). Briefly, poly(lactic-*co*-glycolic acid) (PLGA), preglycated polylactic acid (PLA-PEG), and rapamycin were dissolved in dichloromethane to form an oil phase. The oil phase was then added to an aqueous solution of polyvinyl alcohol and emulsified by sonication (Branson Digital Sonifier 250A). Following emulsification, single emulsions were added to a beaker containing phosphate buffer solution (PBS) and stirred at room temperature for 2 h to allow the dichloromethane to evaporate. The resulting NP were washed twice by centrifuging at 75,600*g* and 4 °C followed by re-suspension of the pellet in PBS. Each SVP-Rapa injection consisted of ~ 50 μg of rapamycin. Methotrexate was purchased from Calbiochem (San Diego, CA, USA). Diphenhydramine was purchased from Baxter Healthcare Corporation (Deerfield, IL, USA).

### 2.2 Mice and treatment

Homozygous GAA-KO mice (6^*neo*^/6^*neo*^), generated by Raben and colleagues by targeted disruption of the *GAA* gene [20], were used in this study. A total of 15 male mice were used for ERT with weekly intravenous injections of 20 mg/kg rhGAA. For each mouse, pretreatment with 15 mg/kg diphenhydramine by intraperitoneal (IP) injection was performed 10–15 min prior to intravenous (IV) administration of rhGAA to prevent anaphylactic reactions [21]. The ERT was initiated at age of 10 weeks (set as ERT week 0) and ended at age of 22 weeks (ERT week 12) and mice received 13 injections of rhGAA in total. These mice were randomly divided into 3 groups (n = 5 each) for different adjunct treatments as described below. Group 1 (Empty NP group): 4 ml/kg empty NP was mixed with rhGAA for injection in ERT weeks 0, 1, and 2; Group 2 (SVP-Rapa group): 4 ml/kg SVP-Rapa was mixed with rhGAA for injection in ERT weeks 0, 1, and 2. Group 3 (MTX group): 3 consecutive IP injections of MTX (10 mg/kg) were given at 0, 24, and 48 h after IV injection of rhGAA in each of week 0, 1, and 2 of ERT, as previously described [21]. All animal experiments were approved by the Institutional Animal Care and Use Committee of Duke University, and following local and national guidelines and regulations.

### 2.3 Sample collection and analyses

Plasma samples were obtained every two weeks 4–6 days following rhGAA administration and stored at − 80 °C for later analysis of anti-rhGAA antibody titer. Urine samples were collected prior to ERT and after 12 weeks of ERT. Total urinary hexose tetrasaccharide (Glca1-6Glca1-4Glca1-4Glc (Glc_4_), Hex_4_) tests were performed for therapeutic responses by liquid chromatography-stable isotope dilution tandem mass spectrometry (LC-MS/MS) as described [22]. Rota-rod tests were performed every 4 weeks to determine motor balance, strength, and coordination [23]. Mice were euthanized 48 h after the last rhGAA injection following overnight fasting. All tissues were kept frozen for evaluating glycogen content and GAA activity as described [23].

### 2.4 Measurement of anti-rhGAA IgG antibody

The anti-rhGAA antibody titer was measured by enzyme linked immunosorbent assay (ELISA) as described [24]. Briefly, 96-well plates (Corning Inc., Corning, NY, USA) were coated overnight at 4 °C with 100 μl per well 5 μg/ml rhGAA. Following washing with 0.05% Tween 20 in PBS, 100 μl per well diluted serum (1:200) were added in duplicates to rhGAA-coated plates and incubated at 37 °C for 1 h. The plates were washed, and alkaline phosphatase-conjugated goat anti-mouse IgG secondary Ab (Cat # 115-055-205, Jackson ImmunoReasearch Laboratory Inc., West Grove, PA, USA) was added and allowed to incubate for 1 h at 37 °C. Following a final wash, 4-Nitronphenyl phosphate disodium salt hexahydrate (Sigma-Aldrich Co., St. Louis, MO, USA) was added and allowed to develop for 20 min at room temperature. Absorbance at 405 nm was read on a VICTOR X Multilabel Plate Reader (PerkinElmer Corporation, Waltham, MA, USA).

### 2.5 Statistical analysis

One-way ANOVA with post hoc test (Tukey) was performed to analyze the differences among the three groups. If the data did not meet the Shapiro-Wilk test for normality, the Kruskal-Wallis test and Mann-Whitney *U* test were performed for nonparametric data. Data in graphs were presented as mean ± standard deviation (SD) or standard errors of mean (SEM) as indicated. The urinary Hex_4_ levels prior to and post ERT were compared using paired *t-*test. Data analyses were conducted using SPSS version 20.0 for Windows (IBM Corp, Armonk, NY, USA), and p < 0.05 was considered significant.

## 3 Results

### 3.1 Immune tolerance induction against rhGAA

**Fig. 1:**
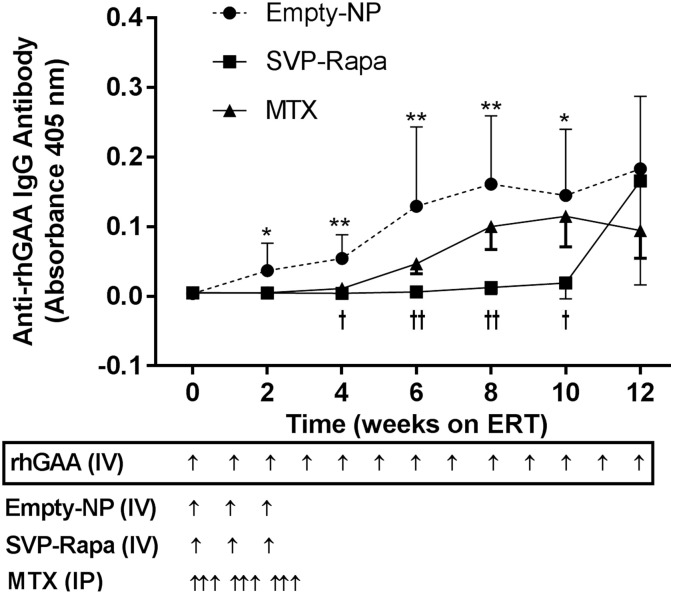
Anti-rhGAA antibody titers in GAA KO mice treated with three different regimens. Naive GAA-KO mice (age of 10 weeks) received weekly intravenous injection of 20 mg/kg of rhGAA (ERT) for 12 weeks plus one of the three adjunct treatments: empty NP (n = 5), SVP-Rapa (n = 5), or MTX group (n = 5). Details of the treatments are described in Material and methods. Anti-rhGAA antibody levels were assessed by ELISA using 1:200 diluted plasma samples. Data were presented by the absorbance at 405 nm (mean ± SD) and analyzed by one-way ANOVA with post hoc test (Tukey). For Week 0, n = 15 (all mice); for other weeks on ERT, n = 5 for each group. *p < 0.05, **p < 0.01 (comparison between SVP-Rapa and Empty NP); ^†^p < 0.05, ^††^p < 0.01 (comparison between SVP-Rapa and MTX).

Co-administration of SVP-Rapa with the first three doses of rhGAA effectively prevented anti-rhGAA antibody development throughout the 12-week study period except for ERT week 12 (Fig. 1). After 12 weeks on ERT, two of the five mice in the SVP-Rapa group showed an increase of anti-rhGAA antibody, while the remaining three animals showed no sign of antibody formation. The empty NP co-treatment did not show any suppressive effect on anti-rhGAA antibody response, as the kinetics of anti-rhGAA antibody in the Empty NP group was similar to that in GAA-KO mice on ERT with rhGAA only as reported previously [21], [25]. Mice treated with MTX at 0, 24, and 48 h after each of the first three injections of rhGAA started developing anti-rhGAA antibody from ERT week 6, and the overall antibody titers in the MTX group were lower than those in the Empty NP group, but higher than those of the SVP-Rapa group except at week 12.

### 3.2 Effects of adjunct treatments on rhGAA uptake and glycogen clearance

**Fig. 2:**
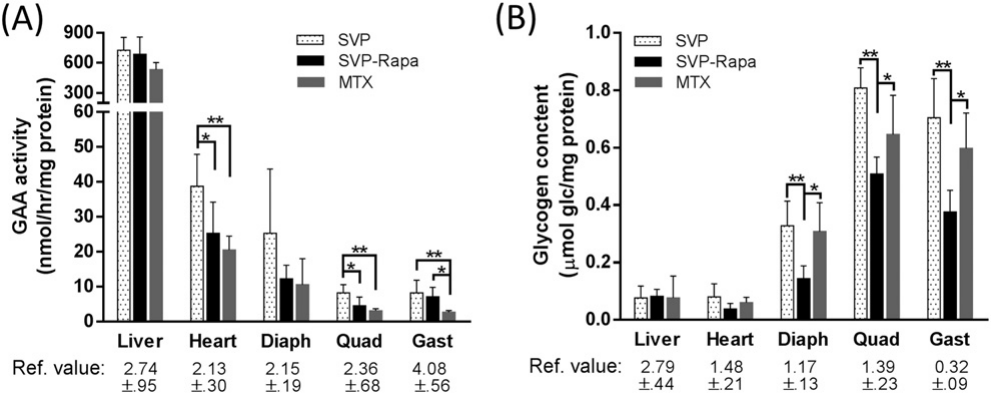
Comparison of GAA enzyme activity (A) and glycogen contents (B) in GAA-KO mouse tissues after treatment with different ERT regimens. Male GAA-KO mice were treated with rhGAA (20 mg/kg, weekly, IV) for 12 weeks plus either empty NP (n = 5, IV), or SVP-Rapa (n = 5, IV), or MTX (n = 5, IP). Values were shown as mean ± SD and analyzed by one-way ANOVA with post hoc analysis (Tukey). *p < 0.05, **p < 0.01. Ref. value, values measured from 7 untreated mice at an age matching the starting age (Week 0) of the mice in the experiment, shown as mean ± SD.

Liver had extremely high GAA activity (533–729 mmol/h/mg) in all three groups of mice on ERT compared with basal activity in GAA-KO mice measured in our laboratory (~ 3 mmol/h/mg), and GAA activity in heart (21–38 mmol/h/mg) was also significantly higher than basal level (~ 2 mmol/h/mg), while uptake of rhGAA by skeletal muscles was poor (Fig. 2A). Among the three groups, the Empty NP group surprisingly demonstrated the highest GAA activities in all tissues despite developing the highest anti-rhGAA antibodies, while the MTX group had the lowest. The ERT largely cleared the glycogen storage in the liver and heart of all the three groups, indicated by measured glycogen content (~ 0.1 μmol Glc/mg in liver and 0.05–0.1 μmol Glc/mg in heart) (Fig. 2B), compared with ~ 2.8 μmol Glc/mg in liver and ~ 1.5 μmol Glc/mg in heart of untreated 3-month-old GAA-KO mice observed in our laboratory (shown in Fig. 2B as *Ref. value*). In skeletal muscles, glycogen clearance by ERT was most efficient in the SVP-Rapa group and least effective in the Empty NP group. The higher ERT efficiencies of the SVP-Rapa group in muscles coincided with the lowered tendency of developing anti-rhGAA antibody response (Fig. 1, Fig. 2B), but it is surprising that the glycogen clearance did not correlate with GAA activities measured in these tissues (Fig. 2A, B). It should be noted that the glycogen clearance data reflects the cumulative activity of rhGAA over the 12 weeks of therapy, whereas the GAA activity data reflects residual GAA activity from the last dose of rhGAA

### 3.3 Physical and clinical outcomes

**Fig. 3:**
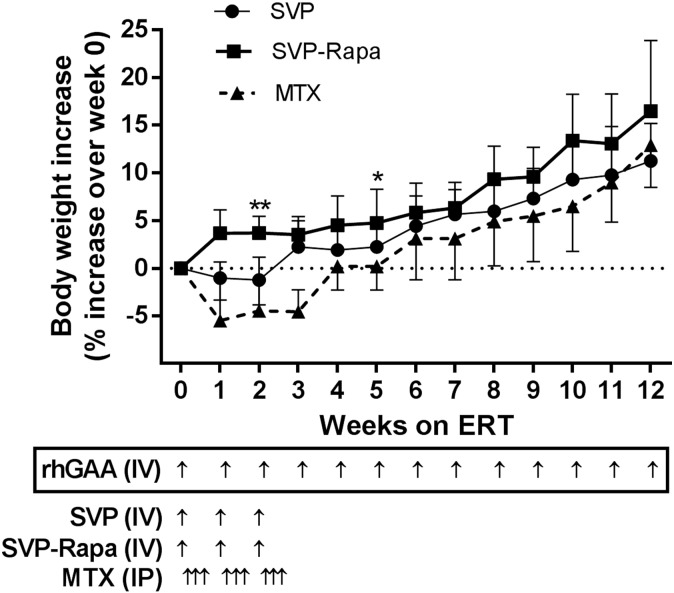
Effect of different adjunct treatment regimens on body weight gain during the12-week course of ERT. Body weight was measured weekly for all mice. Data were presented as percent increase of body weight over the starting weight at Week 0 (mean ± SD) and analyzed by one-way ANOVA with post hoc analysis (Tukey). *p < 0.05 and ** p < 0.01 (SVP-Rapa vs MTX); ^††^p < 0.01 (Empty NP vs MTX).

**Fig. 4:**
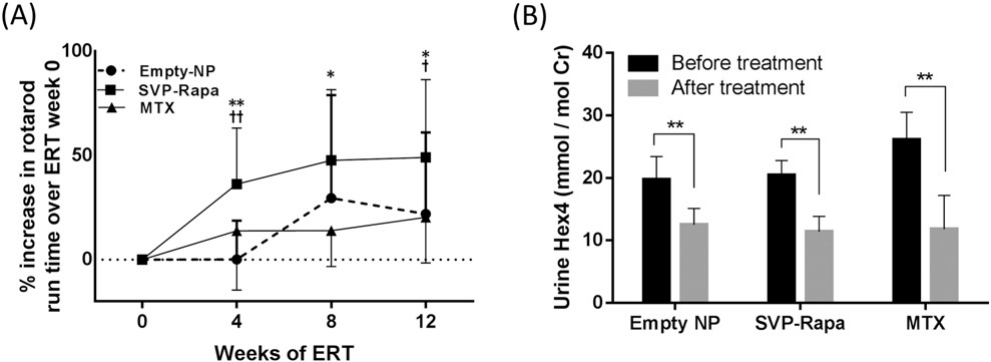
Comparisons of functional benefits following ERT with different adjunct treatment regimens. (A) Improvement of motor function by Rota-rod test. Data were shown as percent change in run time on rod from Week 0 (mean ± SD) and analyzed by one-way ANOVA with post hoc test (Tukey). *p < 0.05 and **p < 0.01 (SVP-Rapa vs MTX); ^†^p < 0.05, ^††^p < 0.01 (SVP-Rapa vs Empty NP). (B) Urinary hexose tetrasaccharide (Hex_4_) content. Data were presented as the mean ± SD and analyzed by paired *t*-tests. **p < 0.01.

Appropriate and steady weight gain is a health indicator in growing animals. A positive effect was observed in the SVP-Rapa group throughout the course of ERT (Fig. 3). In contrast, the MTX-co-treatment exerted a negative effect on growth as indicated by weight loss during the three weeks when MTX was administered (Fig. 3). Improvement in Rota-rod performance (percent increase in fall latency) after 4 weeks on ERT in the SVP-Rapa group was statistically greater than that of the Empty NP group (Fig. 4A). Urinary Hex_4_ levels were significantly reduced in all three groups after ERT, regardless of the adjunct treatment (Fig. 4B).

## 4 Discussion

Enzyme replacement therapy is currently the only effective treatment in patients with Pompe disease (1–3). However, inevitable immune response to ERT with development of HSAT has been a limitation to the injection of the recombinant protein, especially in CRIM negative patients [8], [26], [27]. Several studies have reported that use of immunosuppressant drugs, such as cyclophosphamide, mycophenolate mofetil, belimumab, rituximab, bortezomib, and MTX can lead to successful induction of immune tolerance in GAA-deficient mice and in humans with infantile Pompe disease [9], [10], [11], [12], [13], [21], [27]. Although no serious side effects have been noted in these regimens, concerns about compromised safety due to systemic immunosuppression, reduced cost effectiveness, and the need of long-term treatment still remain.

SVP-Rapa has been demonstrated in several disease models to successfully induce durable antigen-specific immune tolerance and improve functional outcomes [18], [19]. Encapsulation of rapamycin by SVP minimizes its systemic exposure and enhances its uptake by antigen presenting cells, and hence promotes the induction of tolerogenic dendritic cells while avoiding systemic immunosuppression [17], [18], [19]. Here, we evaluated the possibility of adoption of SVP-Rapa as an innovative solution in patients with Pompe disease treated with ERT to induce immune tolerance to rhGAA. The self-assembling, biocompatible, and biodegradable SVP used in this study was made with a synthetic polymer, PLGA, which has been used in a variety of marketed drugs and medical devices [17]. SVP-Rapa has been produced under good manufacturing practice (GMP) conditions and is currently being evaluated in clinical studies in combination with pegsiticase, a highly immunogenic pegylated uricase enzyme for the treatment of refractory gout [28]. Rapamycin, an inhibitor of the mammalian target of rapamycin (mTOR), blocks T-cell activation, inhibits dendritic cells maturation, and selectively allows for stimulation of antigen-specific Foxp3 + regulatory T-cells [29], [30]. Moreover, in GAA-KO mice, rapamycin reduces the accumulation of glycogen via mTOR complex 1 inhibition and increases phosphorylation of glycogen synthase in skeletal muscle [31]. Our study revealed that co-administration of SVP-Rapa with rhGAA has a long-lasting effect on the suppression of anti-rhGAA antibody responses in GAA-KO mice. While three treatments with SVP-Rapa induced durable immune tolerance to ERT, two of the mice developed anti-rhGAA antibodies at 12 weeks after nine challenge injections of rhGAA (Fig. 1). A previous study with coagulation factor VIII (FVIII) in hemophilia A mice have demonstrated that five co-injections of SVP-Rapa with FVIII provided better durability than three co-injections, with tolerance being maintained for at least five months after treatment [17]. Further studies assessing additional co-administrations of SVP-Rapa or a different dose will be required to optimize the regimen for rhGAA.

MTX treatment was used as a positive control in this study because it has been demonstrated that a short-term, low-dose MTX therapy with rhGAA can induce long-lasting immune tolerance to rhGAA in the GAA-KO mouse model [13], [21]. MTX showed good immunomodulatory activity in this study, but four of the five mice showed elevation of anti-rhGAA antibody titers starting from week 6 on ERT. SVP-Rapa has previously been shown to induce more durable induction of immune tolerance than MTX to keyhole limpet hemocyanin (KLH), a highly immunogenic antigen [18].

It has been generally known that the anti-drug antibodies (ADA), when produced in high amounts, could lead to the rapid clearance, degradation, and/or neutralization of enzyme [32], [33]. However, it seems that the anti-rhGAA antibody does not affect the mannose-6-phosphate receptor (M6PR)-mediated enzyme uptake by the liver and muscle cells of GAA-KO mice because our study did not show an enhancement of rhGAA uptake in mice treated with SVP-Rapa or MTX (Fig. 2A). In fact, the GAA activities were higher but glycogen clearance was less efficient in skeletal muscles of the Empty NP treatment group than that of the SVP-Rapa group (Fig. 2A, B). It is possible that the total enzyme activity in the muscles of the empty NP-treated mice is partially contributed by the phagocytic cells (e.g., mast cells, monocytes, and macrophages) in these tissues during the process of Fcγ receptor-mediated endocytosis of the rhGAA-antibody immune complexes [33], [34]. Therefore, the effective GAA activity in muscle cells of the Empty NP-treated mice might be actually lower than that of the SVP-Rapa-treated mice.

Suppression of glycogen synthesis by rapamycin treatment could have contributed to the significantly lower glycogen load in muscles as previously seen in GAA-KO mice and GSD III dogs [31], [35], and this adds to the benefits of using SVP-encapsulated rapamycin as an adjunct treatment. As this study used a mouse model that can be vastly different from humans, clinical investigations will be needed to assess the efficacy of this combined treatment in human patients with Pompe disease.

In summary, our data suggest that co-administration of SVP-Rapa may be an innovative and safe strategy to induce durable immune tolerance to rhGAA during the ERT in patients with Pompe disease.

## Conflict of interest

TKK is an employee and shareholder of Selecta Biosciences. The other authors declare no conflict of interest.

## Funding

This study was supported by a research grant from Selecta Biosciences (to PSK).

## References

[1] Kishnani P.S., Beckemeyer A.A., Mendelsohn N.J. (2012). The new era of Pompe disease: advances in the detection, understanding of the phenotypic spectrum, pathophysiology, and management. Am. J. Med. Genet. C: Semin. Med. Genet..

[2] Hirschhorn R., Reuser A.J.J., Valle D., Scriver C.R. (2009). Glycogen storage disease type II: acid a-glucosidase (acid maltase) deficiency. Scriver's OMMBID the Online Metabolic & Molecular Bases of Inherited Disease.

[3] Kishnani P.S., Hwu W.L., Mandel H., Nicolino M., Yong F., Corzo D. (2006). Infantile-Onset Pompe Disease Natural History Study, a retrospective, multinational, multicenter study on the natural history of infantile-onset Pompe disease. J. Pediatr..

[4] Nicolino M., Byrne B., Wraith J.E., Leslie N., Mandel H., Freyer D.R., Arnold G.L., Pivnick E.K., Ottinger C.J., Robinson P.H., Loo J.C., Smitka M., Jardine P., Tato L., Chabrol B., McCandless S., Kimura S., Mehta L., Bali D., Skrinar A., Morgan C., Rangachari L., Corzo D., Kishnani P.S. (2009). Clinical outcomes after long-term treatment with alglucosidase alfa in infants and children with advanced Pompe disease. Genitourin. Med..

[5] Kishnani P.S., Corzo D., Nicolino M., Byrne B., Mandel H., Hwu W.L., Leslie N., Levine J., Spencer C., McDonald M., Li J., Dumontier J., Halberthal M., Chien Y.H., Hopkin R., Vijayaraghavan S., Gruskin D., Bartholomew D., van der Ploeg A., Clancy J.P., Parini R., Morin G., Beck M., De la Gastine G.S., Jokic M., Thurberg B., Richards S., Bali D., Davison M., Worden M.A., Chen Y.T., Wraith J.E. (2007). Recombinant human acid [alpha]-glucosidase: major clinical benefits in infantile-onset Pompe disease. Neurology.

[6] Banugaria S.G., Prater S.N., Ng Y.K., Kobori J.A., Finkel R.S., Ladda R.L., Chen Y.T., Rosenberg A.S., Kishnani P.S. (2011). The impact of antibodies on clinical outcomes in diseases treated with therapeutic protein: lessons learned from infantile Pompe disease. Genitourin. Med..

[7] Kishnani P.S., Goldenberg P.C., DeArmey S.L., Heller J., Benjamin D., Young S., Bali D., Smith S.A., Li J.S., Mandel H., Koeberl D., Rosenberg A., Chen Y.T. (2010). Cross-reactive immunologic material status affects treatment outcomes in Pompe disease infants. Mol. Genet. Metab..

[8] Berrier K.L., Kazi Z.B., Prater S.N., Bali D.S., Goldstein J., Stefanescu M.C., Rehder C.W., Botha E.G., Ellaway C., Bhattacharya K., Tylki-Szymanska A., Karabul N., Rosenberg A.S., Kishnani P.S. (2015). CRIM-negative infantile Pompe disease: characterization of immune responses in patients treated with ERT monotherapy. Genitourin. Med..

[9] Messinger Y.H., Mendelsohn N.J., Rhead W., Dimmock D., Hershkovitz E., Champion M., Jones S.A., Olson R., White A., Wells C., Bali D., Case L.E., Young S.P., Rosenberg A.S., Kishnani P.S. (2012). Successful immune tolerance induction to enzyme replacement therapy in CRIM-negative infantile Pompe disease. Genitourin. Med..

[10] Elder M.E., Nayak S., Collins S.W., Lawson L.A., Kelley J.S., Herzog R.W., Modica R.F., Lew J., Lawrence R.M., Byrne B.J. (2013). B-cell depletion and immunomodulation before initiation of enzyme replacement therapy blocks the immune response to acid alpha-glucosidase in infantile-onset Pompe disease. J. Pediatr..

[11] Banugaria S.G., Prater S.N., McGann J.K., Feldman J.D., Tannenbaum J.A., Bailey C., Gera R., Conway R.L., Viskochil D., Kobori J.A., Rosenberg A.S., Kishnani P.S. (2013). Bortezomib in the rapid reduction of high sustained antibody titers in disorders treated with therapeutic protein: lessons learned from Pompe disease. Genitourin. Med..

[12] Doerfler P.A., Nayak S., Herzog R.W., Morel L., Byrne B.J. (2015). BAFF blockade prevents anti-drug antibody formation in a mouse model of Pompe disease. Clin. Immunol..

[13] Joly M.S., Martin R.P., Mitra-Kaushik S., Phillips L., D'Angona A., Richards S.M., Joseph A.M. (2014). Transient low-dose methotrexate generates B regulatory cells that mediate antigen-specific tolerance to alglucosidase alfa. J. Immunol..

[14] Cremel M., Guerin N., Campello G., Barthe Q., Berlier W., Horand F., Godfrin Y. (2015). Innovative approach in Pompe disease therapy: induction of immune tolerance by antigen-encapsulated red blood cells. Int. J. Pharm..

[15] Maldonado R.A., von Andrian U.H. (2010). How tolerogenic dendritic cells induce regulatory T cells. Adv. Immunol..

[16] Bachmann M.F., Jennings G.T. (2010). Vaccine delivery: a matter of size, geometry, kinetics and molecular patterns. Nat. Rev. Immunol..

[17] Maldonado R.A., LaMothe R.A., Ferrari J.D., Zhang A.H., Rossi R.J., Kolte P.N., Griset A.P., O'Neil C., Altreuter D.H., Browning E., Johnston L., Farokhzad O.C., Langer R., Scott D.W., von Andrian U.H., Kishimoto T.K. (2015). Polymeric synthetic nanoparticles for the induction of antigen-specific immunological tolerance. Proc. Natl. Acad. Sci. U. S. A..

[18] Kishimoto T.K., Ferrari J.D., LaMothe R.A., Kolte P.N., Griset A.P., O'Neil C., Chan V., Browning E., Chalishazar A., Kuhlman W., Fu F.N., Viseux N., Altreuter D.H., Johnston L., Maldonado R.A. (2016). Improving the efficacy and safety of biologic drugs with tolerogenic nanoparticles. Nat. Nanotechnol..

[19] Zhang A.H., Rossi R.J., Yoon J., Wang H., Scott D.W. (2016). Tolerogenic nanoparticles to induce immunologic tolerance: prevention and reversal of FVIII inhibitor formation. Cell. Immunol..

[20] Raben N., Nagaraju K., Lee E., Kessler P., Byrne B., Lee L., LaMarca M., King C., Ward J., Sauer B., Plotz P. (1998). Targeted disruption of the acid alpha-glucosidase gene in mice causes an illness with critical features of both infantile and adult human glycogen storage disease type II. J. Biol. Chem..

[21] Joseph A., Munroe K., Housman M., Garman R., Richards S. (2008). Immune tolerance induction to enzyme-replacement therapy by co-administration of short-term, low-dose methotrexate in a murine Pompe disease model. Clin. Exp. Immunol..

[22] Young S.P., Zhang H., Corzo D., Thurberg B.L., Bali D., Kishnani P.S., Millington D.S. (2009). Long-term monitoring of patients with infantile-onset Pompe disease on enzyme replacement therapy using a urinary glucose tetrasaccharide biomarker. Genitourin. Med..

[23] Sun B., Zhang H., Franco L.M., Young S.P., Schneider A., Bird A., Amalfitano A., Chen Y.T., Koeberl D.D. (2005). Efficacy of an adeno-associated virus 8-pseudotyped vector in glycogen storage disease type II. Mol. Ther..

[24] Sun B., Bird A., Young S.P., Kishnani P.S., Chen Y.T., Koeberl D.D. (2007). Enhanced response to enzyme replacement therapy in Pompe disease after the induction of immune tolerance. Am. J. Hum. Genet..

[25] Sun B., Banugaria S.G., Prater S.N., Patel T.T., Fredrickson K., Ringler D.J., de Fougerolles A., Rosenberg A.S., Waldmann H., Kishnani P.S. (2014). Non-depleting anti-CD4 monoclonal antibody induces immune tolerance to ERT in a murine model of Pompe disease. Molecular Genetics and Metabolism Reports.

[26] de Vries J.M., van der Beek N.A., Kroos M.A., Ozkan L., van Doorn P.A., Richards S.M., Sung C.C., Brugma J.D., Zandbergen A.A., van der Ploeg A.T., Reuser A.J. (2010). High antibody titer in an adult with Pompe disease affects treatment with alglucosidase alfa. Mol. Genet. Metab..

[27] Banugaria S.G., Patel T.T., Mackey J., Das S., Amalfitano A., Rosenberg A.S., Charrow J., Chen Y.T., Kishnani P.S. (2012). Persistence of high sustained antibodies to enzyme replacement therapy despite extensive immunomodulatory therapy in an infant with Pompe disease: need for agents to target antibody-secreting plasma cells. Mol. Genet. Metab..

[28] Hershkovitz E., Forschner I., Mandel H., Spiegel R., Lerman-Sagie T., Anikster Y., Zeharia A., Moses S. (2014). Glycogen storage disease type III in Israel: presentation and long-term outcome. Pediatr. Endocrinol. Rev..

[29] Li X., Li J.J., Yang J.Y., Wang D.S., Zhao W., Song W.J., Li W.M., Wang J.F., Han W., Zhang Z.C., Yu Y., Cao D.Y., Dou K.F. (2012). Tolerance induction by exosomes from immature dendritic cells and rapamycin in a mouse cardiac allograft model. PLoS One.

[30] Wang C., Yi T., Qin L., Maldonado R.A., von Andrian U.H., Kulkarni S., Tellides G., Pober J.S. (2013). Rapamycin-treated human endothelial cells preferentially activate allogeneic regulatory T cells. J. Clin. Invest..

[31] Ashe K.M., Taylor K.M., Chu Q., Meyers E., Ellis A., Jingozyan V., Klinger K., Finn P.F., Cooper C.G., Chuang W.L., Marshall J., McPherson J.M., Mattaliano R.J., Cheng S.H., Scheule R.K., Moreland R.J. (2010). Inhibition of glycogen biosynthesis via mTORC1 suppression as an adjunct therapy for Pompe disease. Mol. Genet. Metab..

[32] Cousens L.P., Mingozzi F., van der Marel S., Su Y., Garman R., Ferreira V., Martin W., Scott D.W., De Groot A.S. (2012). Teaching tolerance: New approaches to enzyme replacement therapy for Pompe disease. Hum Vaccin Immunother.

[33] Chirmule N., Jawa V., Meibohm B. (2012). Immunogenicity to therapeutic proteins: impact on PK/PD and efficacy. AAPS J..

[34] Nimmerjahn F., Ravetch J.V. (2008). Fcgamma receptors as regulators of immune responses Nature reviews. Immunology.

[35] Yi H., Brooks E.D., Thurberg B.L., Fyfe J.C., Kishnani P.S., Sun B. (2014). Correction of glycogen storage disease type III with rapamycin in a canine model. J. Mol. Med..

